# Sustainable synthesis of nano-crystalline and heterostructural Zr MOF@Bentonite composite, as a novel adsorbent for methylene blue adsorption

**DOI:** 10.1038/s41598-025-01787-5

**Published:** 2025-05-17

**Authors:** Fatma M. Dardir, Ezzat A. Ahmed

**Affiliations:** Geology Department, Faculty of Science, Assiut University, Asyut, Egypt

**Keywords:** Zirconium, Bentonite, MOF, Composite, MB dye, Adsorption, Kinetics, Isothermal models, Thermodynamic studies, Environmental sciences, Materials science

## Abstract

Creating high-performance materials that are abundant in nature is an important area of current research to satisfy the growing need for sustainable, high-availability, low-cost functional adsorbents for industrial pollution removal systems. The natural bentonite collected from Abu Tartur area, Western Desert, Egypt is used as a precursor in the synthesis of ZrMOF@Bentonite composite. The synthesized material was characterized by X-ray diffraction (XRD), scanning electron microscope (SEM), Fourier transformer infrared (FT-IR), and nitrogen sorption at liquid nitrogen temperature. The already prepared adsorbent was used in the removal of methylene blue dye from polluted water. Various impacts were discussed viz., adsorbent dose, solution pH, contact time, and initial concentration of the adsorbate in order to access the equilibrium criteria. The maximum removal reached 99.8% when a pH value equal to 5 and 0.17 g of adsorbent was used, While at pH = 6, the % removal was 90%. Moreover, the maximum adsorption capacity was found to be 13.7 mg/g at time 180 min. This result goes parallel with the calculated q_e_ one. The inspected kinetic models were examined in order to arrive at the adsorption mechanism. The overall different kinetic models were examined and have the best value of R^2^ ≥ 0.96 indicating the adsorption process proceeds through a physisorption nature. The experiment data were analyzed by Langmuir and Freundlich isothermal models exhibiting a high correlation coefficient belonging to the two models (R^2^ ≥ 0.96). The maximum experimental q_e_ is 29 mg/g which matches very well with the theoretically calculated one (q_max_ 32.28 mg/g).

## Introduction

The energy crisis and global environmental pollution represent major challenging issues that affect many aspects in our daily life. Heavy metals, pesticides, antibiotics, dyes, and other dangerous organic pollutants have been impeded to water bodies, as a result of unwise uses and industrialization^1,2^. Colouring dyes and chemical materials used in many fields, such as food, textile, and cosmetic industries for coloring purposes^3^, dumped in water prevent aquatic plants from completing the photosynthesis process, and prevent the entry of flying sunlight^3–5^. Additionally, such exposure to industrial dyes in wastewater may cause several health risks, such as cancer, endocrine disruption, skin and respiratory disorders, reproductive development abnormalities, and may be even neurotoxic. Due to the carcinogenic properties of some dyes, and their by-product, the prolonged exposure may increase the chance of propers cancer. Also, industrial dyes hazards may reach anomalies or fertility issues, hence, productive system’s health is also at risk^6^. Furthermore, Colours containing substances that can irritate the skin, causing allergies when they come into contact with it; breathing in airborne particles or vapors can also cause respiratory problems^7^.

Several techniques are responsible for viz. extracting dyes produced from industry in wastewater. One method for dye removal is chemical precipitation through the addition of some chemicals to form insoluble complexes. This process produces dye precipitation, coagulation, and flocculation, which use agents to produce larger flocs, that are easily removed. Physical methods for eliminating dye particles from wastewater include adsorption onto materials, such as activated carbon and membrane filtering. To break down or absorb dyes, biological methods employ microorganisms for biodegradation or plants for phytoremediation. Advanced oxidation techniques like ozonation and UV photolysis use strong oxidants to change dyes into a less harmful metabolites^8^. Additionally, electrochemical methods such as ion exchange and electrocoagulation provide efficient techniques of colour removal. Selecting the optimal strategy often requires many integrating ways, for a comprehensive and effective wastewater treatment, depending on the dye’s specificity, the desired treatment outcomes, and budgetary considerations^9^. Today, several available and low-cost materials are used as adsorbents to remove dye from wastewater, including phosphates^10,11^, carbonaceous adsorbents^12,13^, clay^14–16^, hydroxyapatite^17^, and zeolites^18^.

Magnetic metal-organic frameworks (MOFs) are clearly very active and promising way to remove dye from wastewater. These MOFs have a high adsorption capacity, because of their large surface areas and distinct porous architectures. These are distinguished well by their magnetic responsiveness, which is due to the presence of magnetic components^19^. This feature streamlines the recovery process and excludes the need for complex separation procedures by enabling the quick and simple separation of MOFs from treated water, using an external magnetic field. The reusability and renderability of magnetic MOFs are further demonstrated by the fact that they may be easily recovered and often undergo desorption procedures to restore their adsorption capacity^20^. The efficiency of the process is increased due to their fast adsorption kinetics, adaptable properties, and dye molecule selectivity. Because this approach is scalable and adaptable enough to handle varying wastewater volumes, it is utilized in a variety of industrial applications. It is worth mentioning that, the use of magnetic MOFs in dye removal not only shows an effectiveness but also contributes to a reduction in environmental impact by preventing the creation of secondary waste. As research and development in this area will be in progress, therefore magnetic MOFs hold great promise for environmentally friendly and effective wastewater treatment solutions^21^.

Recently, mesoporous silica materials have been widely used for the treatment of contamination in water. However, with the increasing consumption of such types of materials containing silica for large-scale industrial applications, the costs of the above-mentioned materials utilizing commercial silica precursors are relatively high^22^. Therefore, it is highly desirable to realize the production of several bentonites (silica-containing) as another source of silica by employing such cheaper precursors.

In the present work, the natural, low-cost, and available bentonite is used as a precursor in the synthesis of a new porous material from the metal-organic framework group (ZrMOF@Bentonite). The combination of zirconium, MOF, and bentonite is used for the first time. Zr/bentonite- MOF has a high surface area, and a high capability to eliminate the dye from polluted water. So, it is used as an efficient agent for the adsorption of methylene blue dye. Therefore the impacts of the adsorbent dose, solution pH, contact time, and initial concentration of MB dye are studied. Additionally, some kinetics and isothermal models are studied, in order to complete the criteria of the adsorption mechanism.

## Experimental work

### Materials

In the present work, raw bentonite was collected from Abu Tartur area, Western Desert, Egypt. Zirconium oxychloride (ZrOCl_2_.8H_2_O) (99% sigma aldrich), benzene dicarboxylic acid (BDC) (98% alfa ether), dimethyl formamide (DMF) (99.5% alfa chemicals), Acetic acid, and ethanol (99%) are used in the synthesis process. All chemicals used are of a high grade. Methylene blue dye was used in the adsorption experiment. Sodium hydroxide (0.1 M) and nitric acid (Aldrich, 70%) are used to follow the pH of the solution.

### ZrMOF@Bentonite synthesis

To synthesize ZrMOF@bentonite, the raw sample (bentonite) was crushed into a powder. 0.5 g of bentonite was dissolved in 25 ml distilled water. Subsequently, 1.8 g of zirconium oxychloride was added to the former solution, and sonicated for 5 min (mixture 1). Then 0.987 g of benzene dicarboxylic acid (BDC) was dissolved in 120 ml dimethyl formamide (DMF) and sonicated for 5 min (mixture 2). In the next step, mixture 1 was added to mixture 2 to form the mixture 3. Acetic acid (5.3 ml) was added to the last mixture and sonicated for 5 min. The mixer was transferred to the oven for one hour at 120 °C, followed by steering for overnight. Finally, the product was washed with methanol several times and separated using a centrifuge and dried at 70 °C for 24 h.

### Characterization technique

XRD is a technique used to study the mineral composition of the raw material and the synthesized product. High-energy, sensitive X-ray radiation. XRD patterns achieved by measuring the (2θ/ degree) at which the sample diffracts an X-ray beam of wavelength (λ) and intensity (I). The JSD-60/ Joel diffractometers (Japan) with Ni-filtered Cukα radiation at Assiut University, were used for XRD analysis of the current investigated samples. They operated at 20 mA and 40 kV in the 2θ range of 4 to 60 2θ with a scanning rate of 5°/min. The morphology of synthesized materials was studied by using the scanning electron microscope model JSM-5400 LV (Jeol, Tokyo, Japan). A 6700 Nicolet Spectrophotometer (USA) was used to identify the chemical functional groups in the studied sample. The material’s spectra were recorded in the 4000–400 cm^−1^ range. Nitrogen adsorption/desorption measurements were carried out, using a micrometrics instrument model ASAP 2010 (USA) at 196 °C. Prior to analysis, the samples were degassed at 110 °C under vacuum conditions for 3 h. The adsorption data were processed by the instrument software to obtain the specific surface area by the BET equation. The texture analysis of the materials was determined via the t-plot method using the Haisey equation. Total pore volume Vt was calculated at P/P° = 0.95. Pore volume distributions were calculated by BET (PBET) and BJH (PBJH) methods.

### Adsorption experiment

In the adsorption experiment of MB dye, 1000 mg/l of a standard solution stock was prepared. Four factors were studied dose, pH solution, time contact, and initial concentration. In order to examine the effect of the adsorbent dose (0.025, 0.05, 0.07. 0.1, 0.12 0.15, 0.17, 0.2, and 0.22 g) were used, solution pH value = 5, contact time 1.5 h, and 30 mg/l concentration from MB dye. In this study, the effect of the pH solution used pH values ranging from 2 to 9 pH, an adsorbent dose of 0.05 g, a time span of 1.5 h, and a 30 mg/l concentration of MB dye. To calculate the removal percentage the following calculation (1) was used.

1 $$\:\text{R}\text{\%}=\:\frac{\text{C}\text{i}-\text{C}\text{f}}{\text{C}\text{i}}\:\times\:100$$

Where R%, C_i_, and C_f_ are the removal percent, initial concentration, and final concentration respectively.

To study the adsorption capacity at different times, the adsorption achieved through 10, 20, 30, 50, 70, 90, 110, 120, 150, and 180 min, solution pH 5, adsorbent dose 0.05 g, and 30 mg/l concentration of MB dye. The adsorption capacity was calculated from Eq. (2).

2 $$\:qe=\:\frac{\left(Ci-Cf\right)\times\:V}{m}$$

Where q_e_, C_i_, and C_f_, v, and m are the adsorption capacity, initial concentration, and final concentration, volume of solution (ml)n and m adsorbent dose (mg) respectively.

Four kinetic models are investigated in order to examine the effect of contact time viz. intra-particle diffusion, first-perdue order, second-pseudo order, and Elovich models. The various parameters were estimated according to Eqs. (3, 4,5, and 6).

3 $${{\text{q}}_{\text{t}}}={{\text{k}}_{\text{p}}}{{\text{t}}^{1/2}}+{\text{C}}$$

Where Kp is the constant rate of the intra-particle diffusion model (mg/g^− 1^ min ^− 1/2^) and C represents the intercept, related to the thickness of the boundary layer.

4 $$\ln \;\left( {{{\text{q}}_{\text{e}}} - {{\text{q}}_{\text{t}}}} \right)=\ln \;\left( {{{\text{q}}_{\text{e}}}} \right) - {\text{kt}}$$

Where q_e_ is the adsorption capacity at the equilibrium, q_t_ is the adsorption capacity at a given time t, and k is the constant rate given in Eq. (5).

5 $$\:\frac{t}{qt}=\:\left(\frac{1}{{k}^{2}}{q}_{e}^{2}\right)+\hspace{0.17em}\text{t}/{q}_{e}$$

Where q_t_ is the adsorbed amount of methylene blue dye and K_2_ is the constant rate of pseudo-second order uptake (g/mg min).

6 $${{\text{q}}_{\text{t}}}=1/\beta \ln {\text{ }}(\alpha \beta )+1/\beta \ln {\text{ t}}$$

Where α is the initial adsorption rate (mg/g min) at a constant time t = 0 min and β is the degree of activation energy and surface coverage (g/mg).

To study the adsorption capacity at different concentrations, the adsorption was performed through 1.5 h, solution pH 5, adsorbent dose 0.05 g, and (10, 20, 30, 40, 50, 60, 70, 80, 90, 100, and 110) mg/l concentration of MB dye. The adsorption capacity was calculated from Eq. (2). The behavior of the adsorption was studied by using Freundlich and Langmuir models. The parameters of isothermal models were calculated by using Eqs. (7–9).

7 $${C_e}/{q_e}=\left( {1/b{\text{ }}{q_{\hbox{max} }}} \right)+\left( {{C_e}/{q_{\hbox{max} }}} \right)$$

Where C_e_, q_e_, q_max_, and b are the equilibrium concentration of MB dye in the solution, after the adsorption process (mg/l), the uptake capacity per unit mass at equilibrium (mg/g), the maximum amount of adsorbed per unit mass of adsorbent, where monolayer coverage is completed (m mol/g), and the Langmuir constant (L/mg) respectively.

A dimensionless separation factor (R_L_), is determined by Eq. (8), and can be used as a key to characterize the key characteristic of the Langmuir isotherm.

8 $${R_L}={\text{ }}1/\left( {1+b{\text{ }}{C_i}} \right)$$

9 $${\text{Log}}\;{{\text{q}}_{\text{e}}}=\left( {1/{\text{n}}} \right){\text{ }}\log \;{{\text{C}}_{\text{e}}}+\log \;{{\text{K}}_{\text{F}}}$$

Where K_F_ and n are Freundlich constants identified with adsorption capacity and intensity, respectively.

## Results and discussions

### Characterization analysis

#### X-ray diffraction (XRD)

**Fig. 1:**
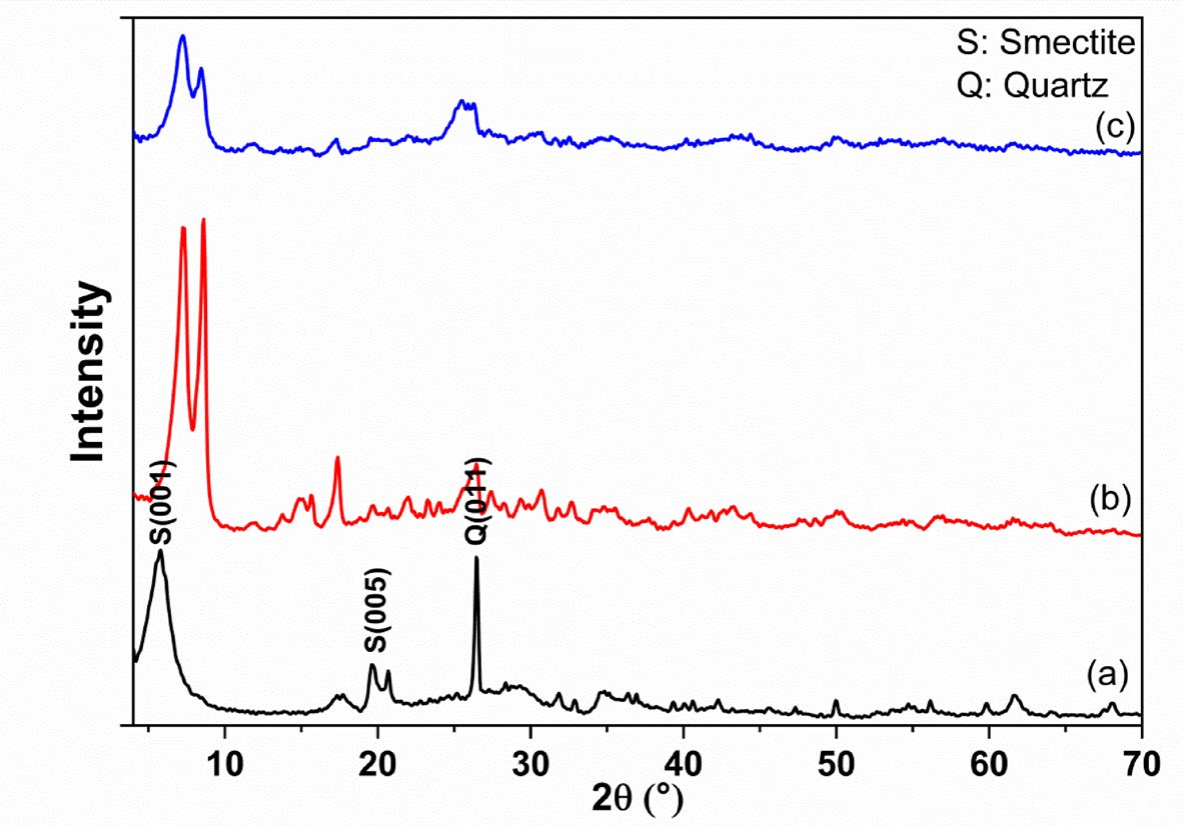
XRD of raw materials (**a**), the synthesized composite before the adsorption (**b**), and the synthesized material after the adsorption process (**c**).

X-ray diffraction pattern of the raw sample is illustrated in Fig. 1a. The pattern shows that the sample is composed of smectite and quartz. The main peaks appear at 2Ɵ = 5.67 and 26.6 of smectite and quartz respectively (49-1498 JCPDS)^23^. The XRD pattern of the synthesized material is shown in Fig. 1b. The pattern revealed that the peaks of smectite and quartz completely disappeared where the two new peaks appeared. These peaks reflect the good crystallinity of synthesized MOF. As indicated from the distinctive two peaks at 7.4° and 8.5°. The synthesized MOF had an isostructural UiO-66 framework topology^24,25^. Figure 1c corresponds to the pattern of the synthesized material after the adsorption process. The pattern revealed that the intensity of the obtained lines decreases where their width increases.

#### Scanning Electron microscope (SEM)

**Fig. 2:**
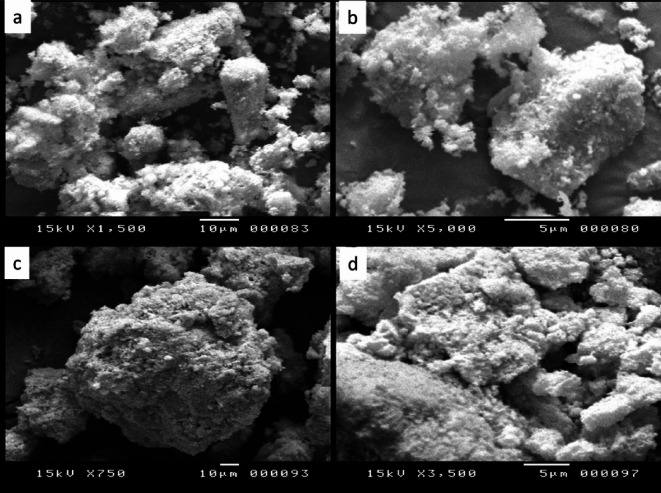
SEM images of the synthesized substance before (**a**,**b**) and after (**c**,**d**) the adsorption process.

The SEM images of the resulting composite using different magnifications before and after the adsorption of MB molecules are seen in Fig. 2. SEM images show that the already synthesized MOF is dispersed and well impeded in the mesostructure phase. Moreover, the figure indicates that it is closely attached together. It is worth mentioning that the bentonite is completely covered by ZrBDC MOF forming a large aggregate in a uniform nature and well distributed in the whole image (Fig. 2a,b). Additionally, these aggregates revealed the observed increase in agglomeration resulting from the adsorption of MB molecules (Fig. 2c,d). Further focusing on the obtained image shows a dark spot even with the high magnification. As depicted in the different images the obtained composite exhibits an irregular shape and worm-like mesostructure. From the above-observed results, we may conclude that these are consistent aforementioned N_2_ adsorption-desorption measurements and low-angle XRD.

#### Fourier-transform infrared (FT-IR)

**Fig. 3:**
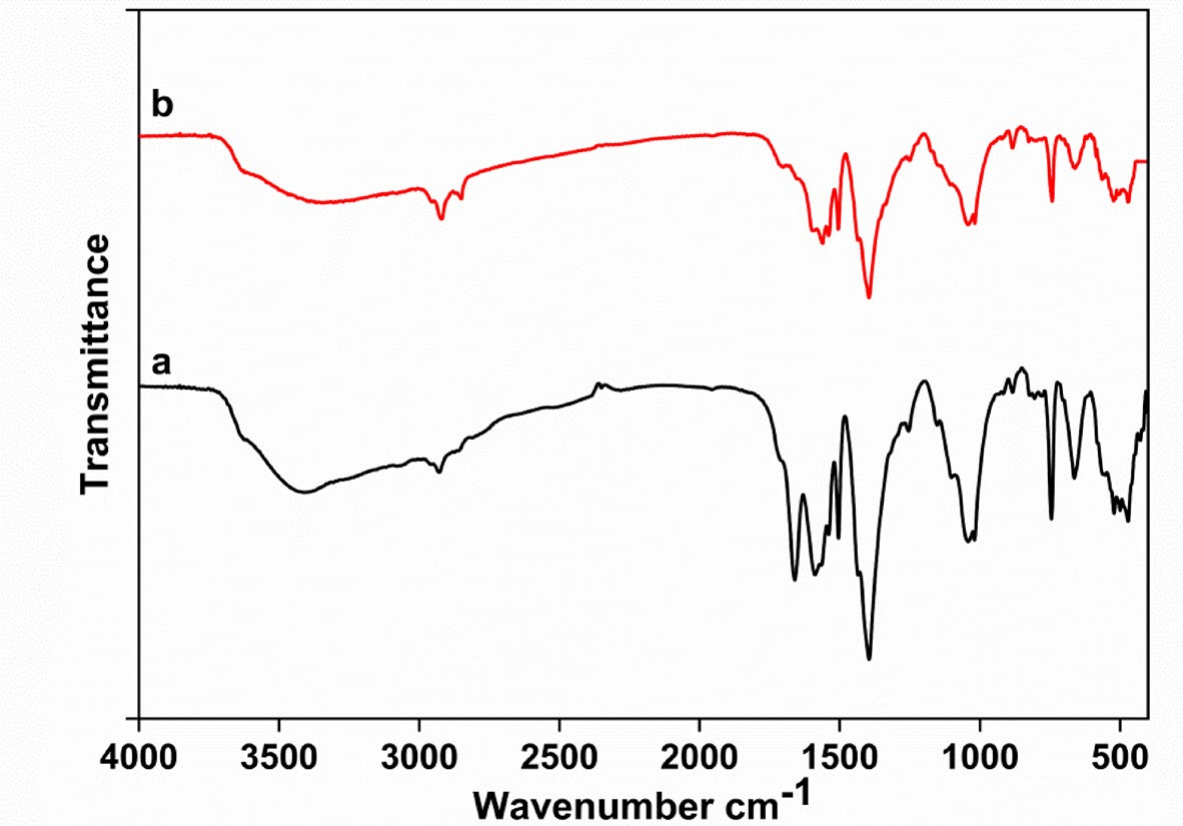
FT-IR of the synthesized material (**a**) and the adsorbent after the adsorption process (**b**).

Fourier transform infrared as measured for probing and confirming the chemical composition of the resulting composite, and the cross-linking between bentonite and filler material, ZrBDC. Inspection of the FT-IR spectrum (Fig. 3), the assignment of the adsorbent pattern before the adsorption indicates a well-distinguished band located at 660, 740, 1015, 1395, 1503, 1585, 1662, and 3418 cm^−1^ shown in Fig. 3a. The band that appeared at 3418 cm^−1^ is related to the O-H group. The intense peak around 1503 cm^− 1^ is attributed to C = C stretch vibrations, while the others at 1015, and 714 can be assigned to C-C bond and C = O stretching vibration respectively^26^. On the other hand, the IR spectrum of the ZrBDC showed a characteristic vibration bands of the benzene ring in the range 1395–1503 cm^−1^ which agrees with the published literature^27^. The intense doublet peaks at 1585 and 1662 may attributed to the in-and-out phases stretching modes of the carboxylate groups, that are present in the terephthalic acid linker^28^. The bands around 740 and 660 cm^−1^ correspond to the O-H and C-H in legend^29^. The spectrum of adsorbent after the adsorption process is shown in Fig. 3b. The spectrum shows the band at 1662 cm^−1^ is disappeared, whereas the band at 1585 cm^−1^ resulted in a noticeable decrease in intensity. These observations can be attributed to the surface coverage belonging to the active sites of the adsorbent which are occupied by MB molecules. These results match very well with Nimbalkar and Bhat 2021^24^.

### Nitrogen adsorption studies

**Fig. 4:**
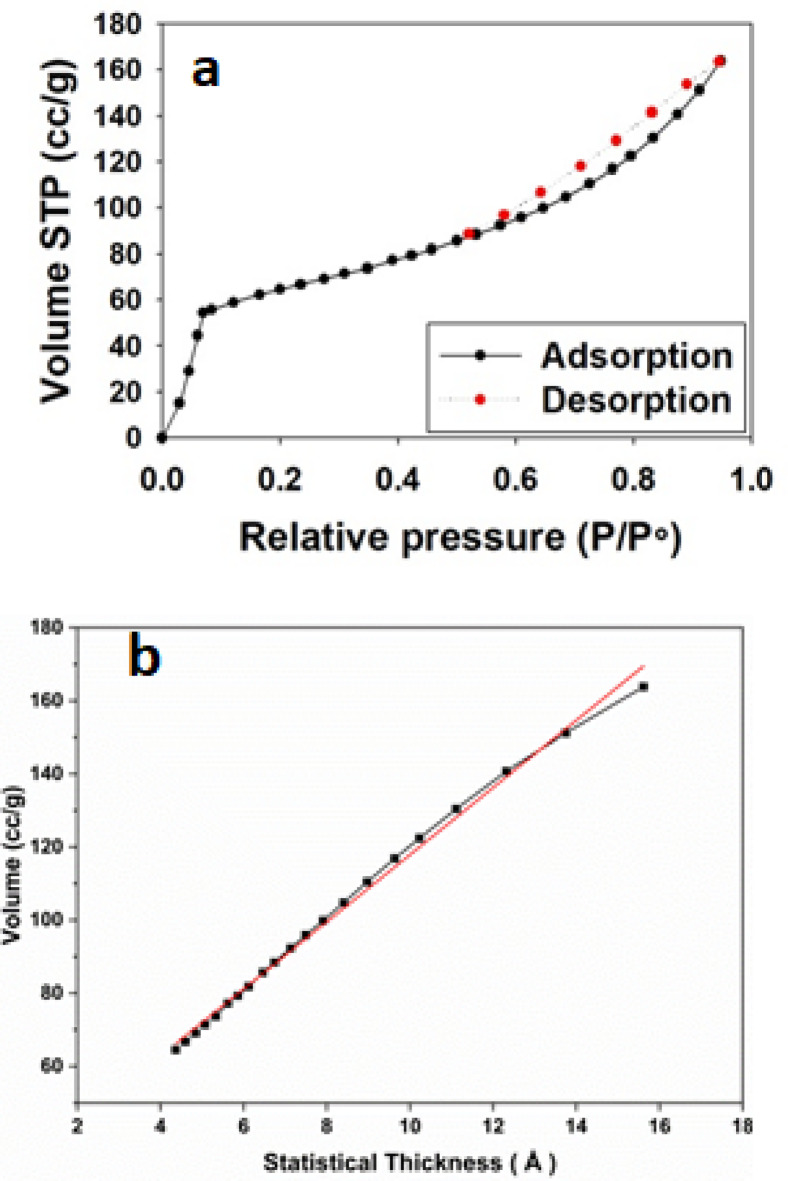

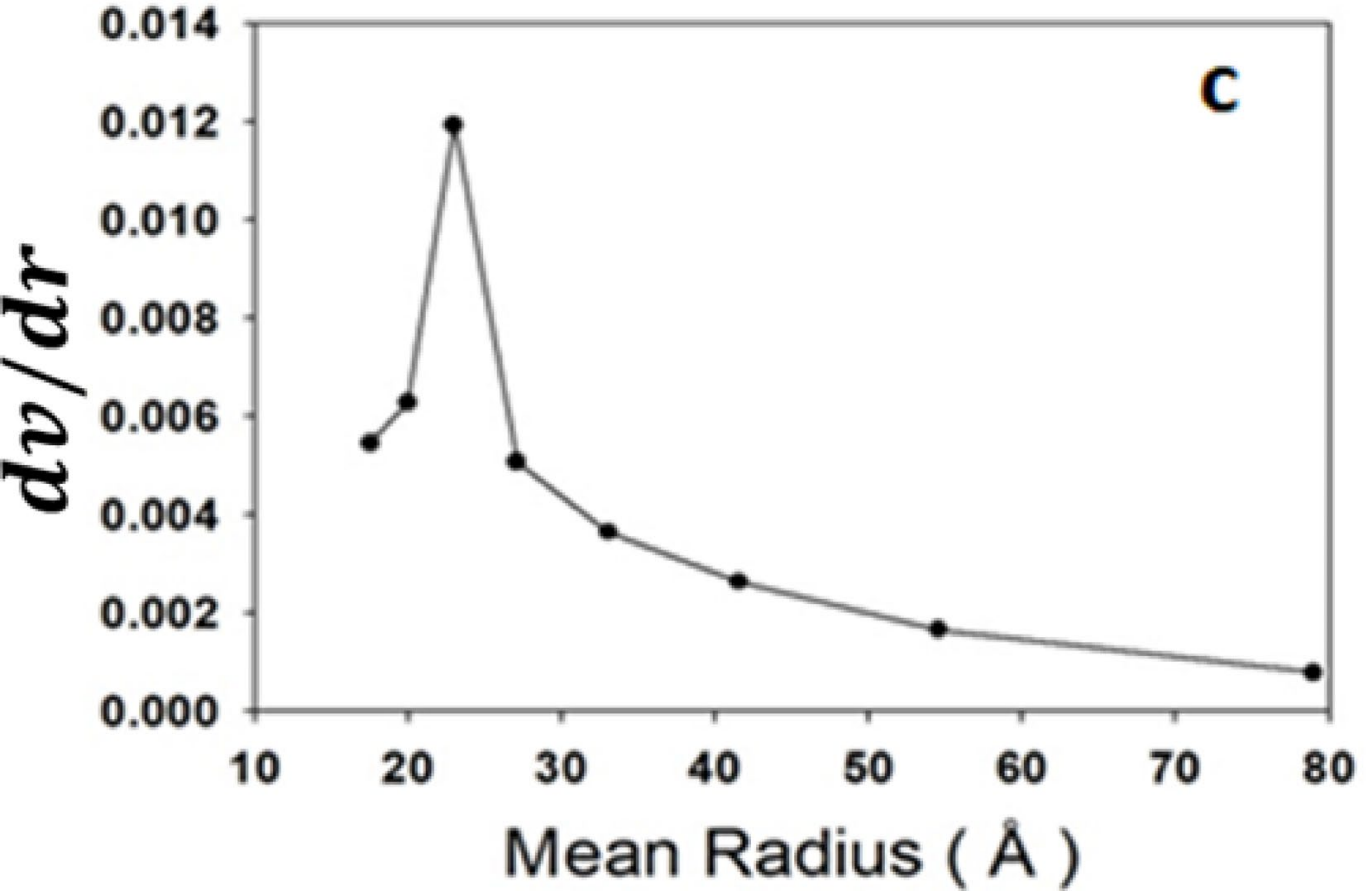
Texture analysis of the adsorbent (**a**) Adsorption-desporption isotherm, (**b**) t-plot, and (**c**) pore volume distribution of ZrMOF@Bentonite.

The nitrogen adsorption-desorption isotherm of the adsorbent is shown in Fig. 4 which indicates that it belongs to an isotherm of a type Ш. Profile as IUPAC classification with H3 hysteresis loop indicating the presence of mesopores^30,31^ as illustrated in Fig. 4a. Applying the BET equation in the range of 0.05–0.35 P/P_0_, the surface area was found to be 212 m^2^/g. Moreover, by analyzing both of the adsorption and desorption branches, we can construct a t-thickness and a pore volume distribution curves (Fig. 4b and c) respectively. The upward deviation in the t carve (b) and the peak located at pore radius > 20 Å in the pore distribution carve (c) confirm the mesopores nature of the adsorbent.

### Adsorption results

#### Effect of adsorbent dose

**Fig. 5:**
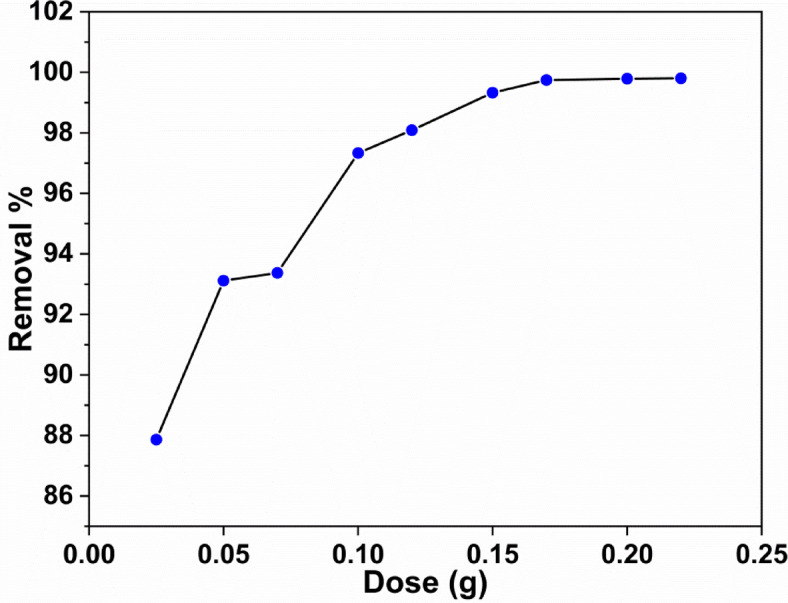
The relation between adsorbent dose and removal percentage.

The relation between the adsorption rate and the adsorbent dose is a critical factor due to the available surface coverage of the adsorbent^32^. So, the relation between the adsorbent dose and the percentage removal of the dye was examined and the data obtained are illustrated in Fig. 5. The figure shows that the adsorption progress proceeded through two stages. The first one indicates that the % removal increases rapidly up to 99.8%, when the adsorbent dose reached 0.17 g. Beyond this dose, the second step becomes a slower before reaching the equilibrium position, where the removal process is 99.8%. This phenomenon can be explained by clarifying the fact that in the first stage, the surface of the adsorbent is bare, and most active sites are available for adsorption^33,34^. Thus, the adsorption forces are quite strong. In the second stage, the active sites are partially occupied and become saturated and hence the adsorbate molecules become difficult to adsorb due to the electrostatic hindrance^35^.

### Effect of solution pH

**Fig. 6:**
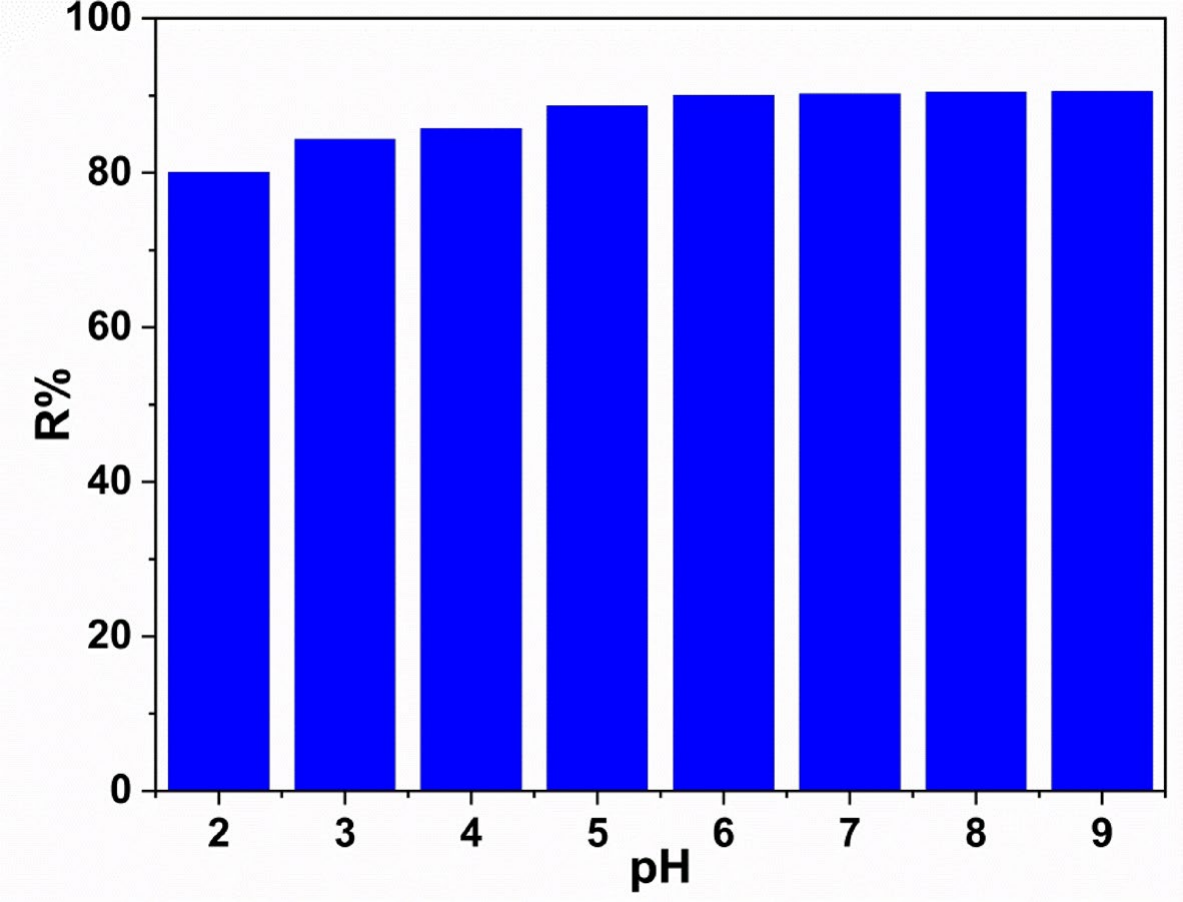
The relation between solution pH and removal percentage.

The solution pH value plays an important role in the adsorption process^18,36,37^. Electrostatic interactions between the ions in the reaction mixture and the adsorption surface are mostly determined the pH of the adsorption mixture. The pH active regions have the potential to impact the nature of the chemical components in the dye solution, as well as the protonation or deprotonation of the adsorbents, where the adsorbate binds^34^. Furthermore, the pH of the solution was examined to identify its effect in the removal percentage. The removal percentage of methylene blue dye is calculated from Eq. (1). The histogram shown in Fig. 6, indicate that by increasing the solution pH will results in a significant enhancement in dye removal. Research on MB has also demonstrated that the elimination efficiency increases as pH rises^35,38^. The curve shows that the removal percentage increases when the solution pH increases. The maximum removal reached 90% at pH value 6. After pH value = 6 the increase in the removal percentage is nearly stable.

It was observed that the initial adsorption efficiency increases gradually with varying the pH value from 2 to 6 which can be interpreted by the electrostatic interaction between the negatively charged sites viz. ZrO and/or SiO_2_, and the positively charged MB molecules. Therefore, the adsorption of such cationic dye, as an adsorbate, proceeds in a such alkaline conditions and retreaded the adsorption process to be optimized at a pH value = 6 often that the adsorption rate becomes slower giving a plateau possesses constant rate.

### Effect of contact time

**Fig. 7:**
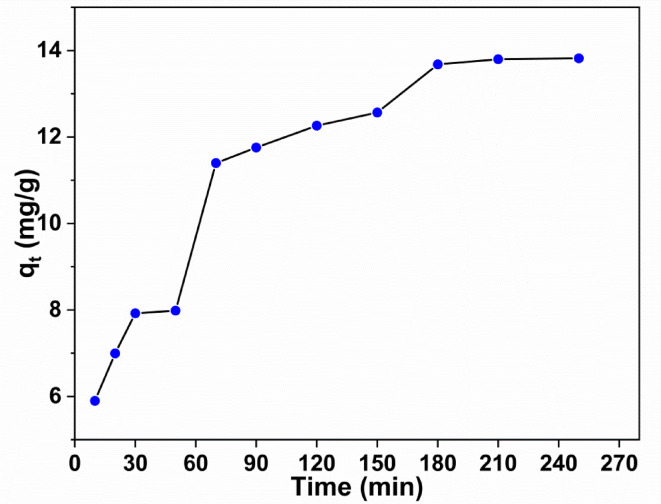
The relation between contact time and adsorption capacity of Zr/Bentonite-MOF.

In order to follow and justify the equilibrium position, it is necessary to study the effect of contact time within the range of 10–180 min for investigating the adsorption process of MB dye^37^. Therefore, the obtained data are illustrated in Fig. 7. Inspection of the figure, it is noticed that the adsorption capacity rapidly increases from 5.8 to 13.7 mg/g at a time up to 180 min. Moreover, the figure shows the trend of variation of qt and time which possess two stages combined by continuous increase in capacity. The first increase can attributed to the partial coverage of the active sites on the adsorbent surface^39^. In the second step, when the contact time increases results in a further increase in q_t_ till we reach a value of 13.7 mg/g at 180 min. This step can be attributed to the saturation of the non-occupied surface sites. Meanwhile, at high times the non-occupied surface sites become more saturated and the rate of adsorption decreases. This circumstance was also mentioned in similar published studies^32,39,40^.

#### Kinetic studies

**Fig. 8:**
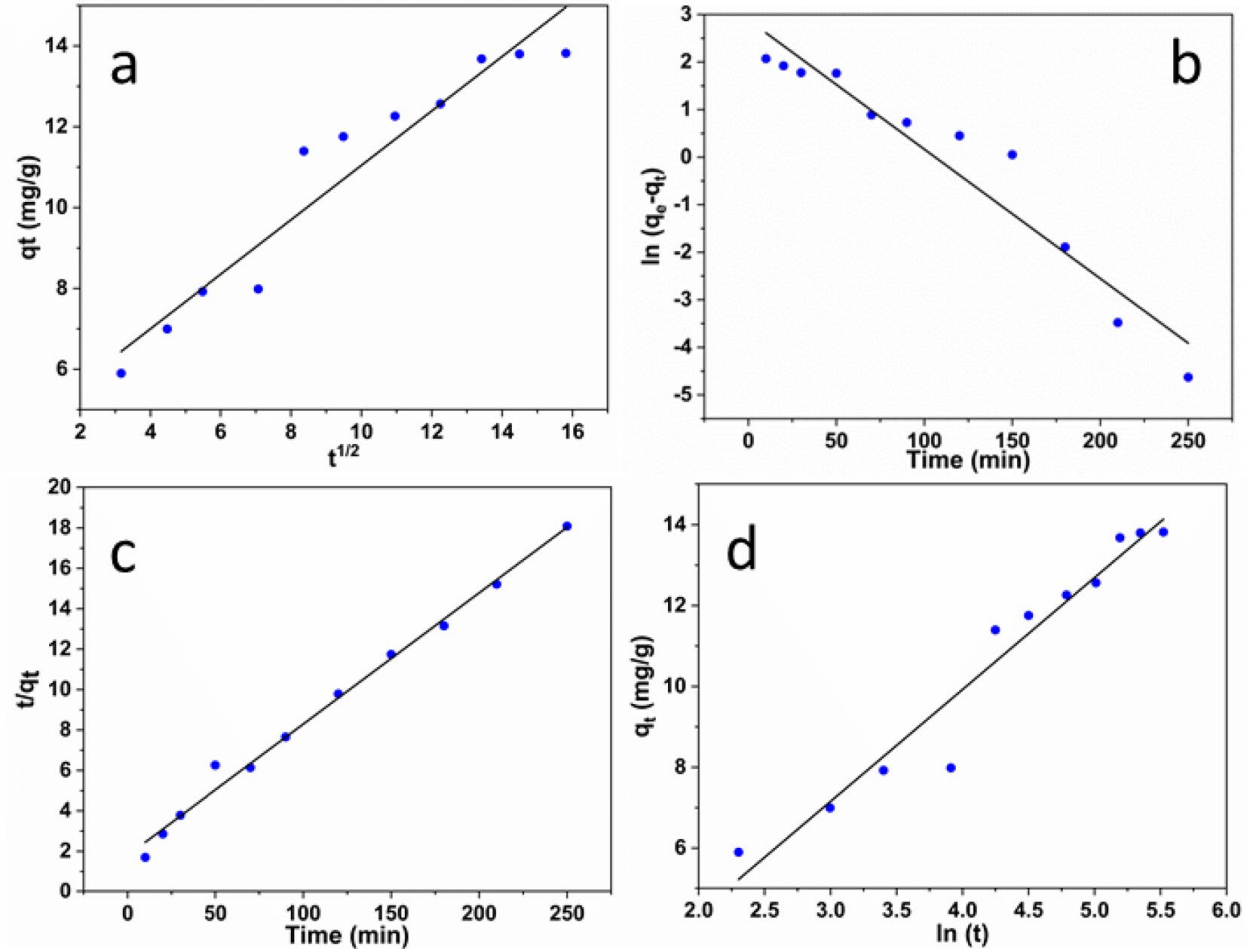
The kinetic models are (**a**) intra-particle diffusion, (**b**) first-pseudo order, (**c**) second-pseudo order, and d) Elovich models.

The adsorption kinetic studies will provide information about the following: (1) the adsorption rate; (2) the adsorption mechanism of mass transform and (3) the adsorbent- adsorbate interaction at different times^40,41^. Moreover, this study demonstrates how forecast quickly pollutants would be eliminated by a solid material, which represents an important consideration in operational design^42^. In this investigation, four kinetic models were applied viz., intra-particle diffusion, pseudo-first order, pseudo-second order, and Elovich models. The obtained kinetic data are treated by using Eqs. (3–6) and the calculated various kinetic parameters are illustrated in Fig. 8 and summarized in Table 1. The appropriate model that could be applied to describe the movement of dye molecules from its aqueous solution tested also the linear regression of such a model and represented in Fig. 8(a-d). The first inspection allover the different models shows a high correlation coefficient values close to unity. It is worth mentioning that the sequence of variation of R^2^ is a follow of pseudo-second-order > Elovich > pseudo-first-order ≥ intra-particle. For the intra-particle diffusion model, it interprets the movement of MB molecules to the adsorbent surface^11,43^ whereas the Elovich model possesses a linear regression presented in Fig. 8d. This model represents the energetic heterogeneous adsorption at the surface of the adsorbent^36^. Finally, the above comparative study reveals that the calculated parameters including its R^2^ fit clearly with the pseudo-second-order kinetic. This model indicates that the adsorption event belongs to the physical isotherm behavior, via both hydrogen bonding and electrostatic sharing between the adsorbent and the dissolved MB dye vid-infra the adsorption mechanism.

**Table 1:** The calculated parameters of kinetic models.

	Parameters	MB dye
Intra-particle diffusion	K_p_ (mg g^− 1^min^0.5^)	0.6734
	C (mg g^− 1^)	4.3087
	R^2^	0.96
Pseudo-frist order	k (min^− 1^)	-0.02719
	q_e_ calc.(mg/g)	17.92
	R^2^	0.96
	q_t_ expr.(mg/g)	13.7
Pseudo-second order	qe (mg/g)	11.76
	k_2_ (mg/min)	4 × 10^− 3^
	R^2^	0.99
Elovich	β (g/mg)	0.3612
	α (mg/g min)	0.1146
	R^2^	0.97

### Effect of initial concentration

**Fig. 9:**
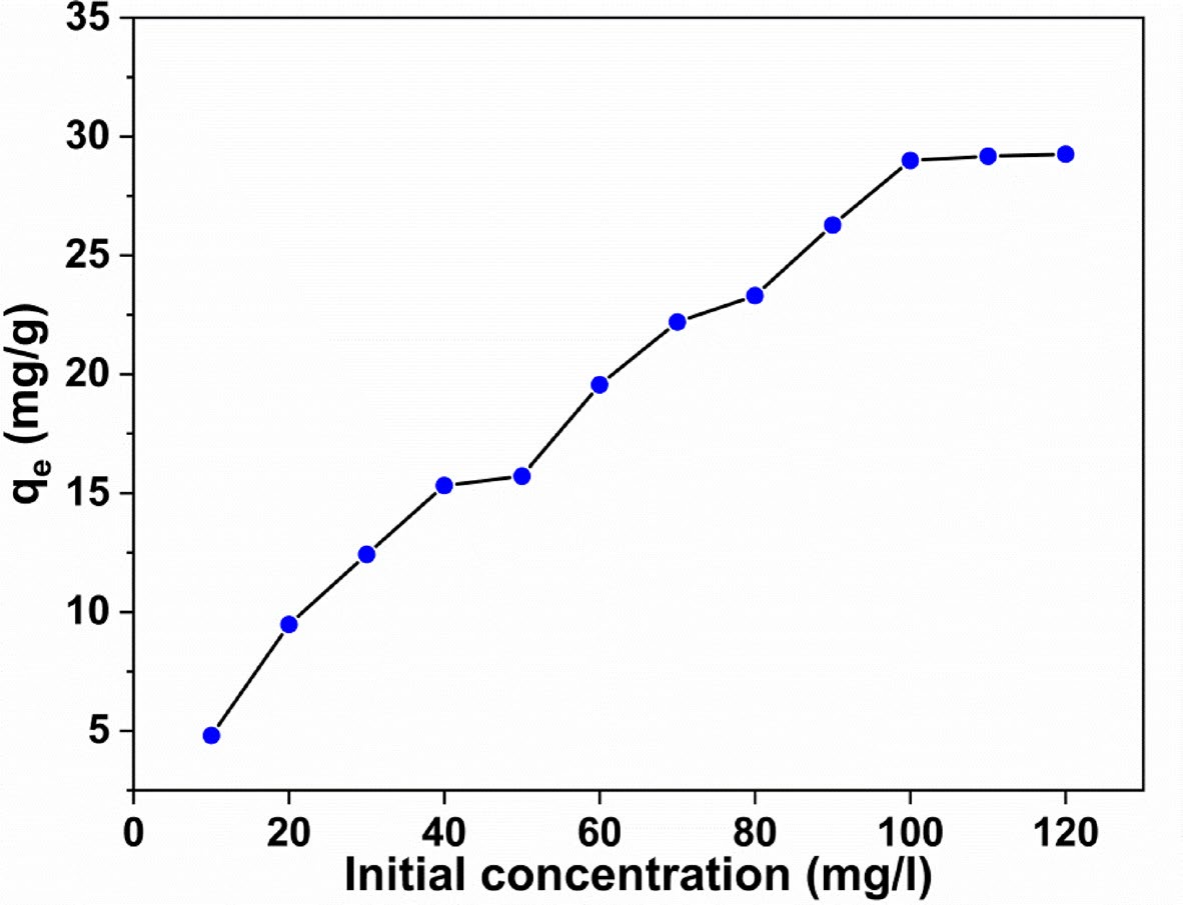
The relation between adsorption capacity and initial concentrations.

The impact of the initial concentration of the adsorbate is a very decisive factor in determining the adsorption capacity of methylene blue dye. Therefore, the results obtained showing in Fig. 9 give insight view of such variation. The curve shows that when the initial concentration increases from 10 mg/l to 100 mg/l it results in a pronounced increase in the capacity of adsorption from 4.7 mg/g to 28.9 mg/g. On the other hand, when it reaches 100 mg/l from the adsorbate, there is a noticeable decrease in capacity, which sheds the light which confirms that condition of the equilibrium state is attained.

The transport of the adsorbate molecules from the solution to the adsorbent surface can be thought of as being a result of the increase in the initial concentration, which in turn, raises the mass gradient. As a result, the attained equilibrium at the surface of the composite is the responsible for the propagation of the adsorbate-adsorbent interaction.

#### Adsorption isotherms

In order to visualize the criteria for the adsorption of MB onto the adsorbent surface of the ZrMOF@Bentonite catalyst, four different isothermal models including Langmuir, Freundlich, Temkin, and Dubinin models were investigated. The Langmuir isotherm is the most common model to discuss the adsorption of organic dye which assumes that the adsorption molecules are arranged in a homogeneous monolayer^44^. Conversely, the Freundlich model assumes the formation of a heterogeneous adsorbed layer^45^. Furthermore, Temkin model takes into account the interaction between adsorbent and adsorbate. Meanwhile, this model sheds light on the heat of adsorption.

**Fig. 10:**
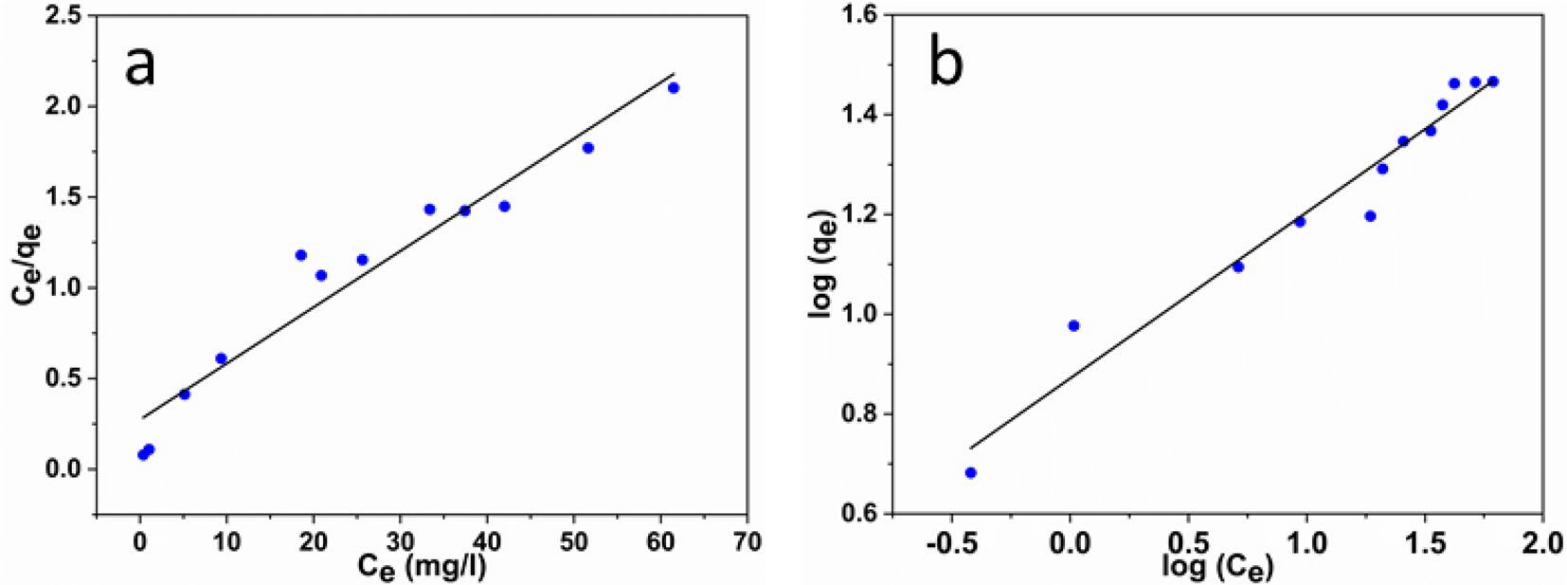
The isothermal models (**a**) Langmuir and (**b**) Freundlich.

The linear isotherms of MB adsorption are plotted in Fig. 10a and b, and the related parameters are listed in Table 2. Based on the linear regression coefficient R^2^ values, the eventual course of adsorption can be best presented by the Langmuir model. This suggests that the adsorption proceeds at specific homogeneous sites and follows a monolayer adsorption event. In addition, the separation factor R_L_ given in Langmuir can be employed to evaluate the feasibility of adsorption^46^.

The evaluated R_L_ value in our study is 0.0812, indicating a favorable adsorption process. It is worth noting, that the Dubinin (D-R) model was applied to estimate the apparent energy of adsorption. Moreover, it is usually used to distinguish the type of adsorption. The calculated Polanyi characteristic constant mean free energy^47^ as appeared from our results has a value ~ 2.69 KJ mol^− 1^, which is below 8 KJ/mol, thus indicating the adsorption underwent a physical process^47^.

**Table 2:** The estimated parameters of isothermal models.

	Parameters	MB dye
Langmuir model	q_max_ (mg/g)	32.28
	b (L/mg)	0.1131
	R^2^	0.97
	q_e_ expr.(mg/g)	28.9
	R_l_	0.0812
Freundlich	1/n	0.333
	K_f_	7.437
	R^2^	0.97

#### Thermodynamic studies

**Fig. 11:**
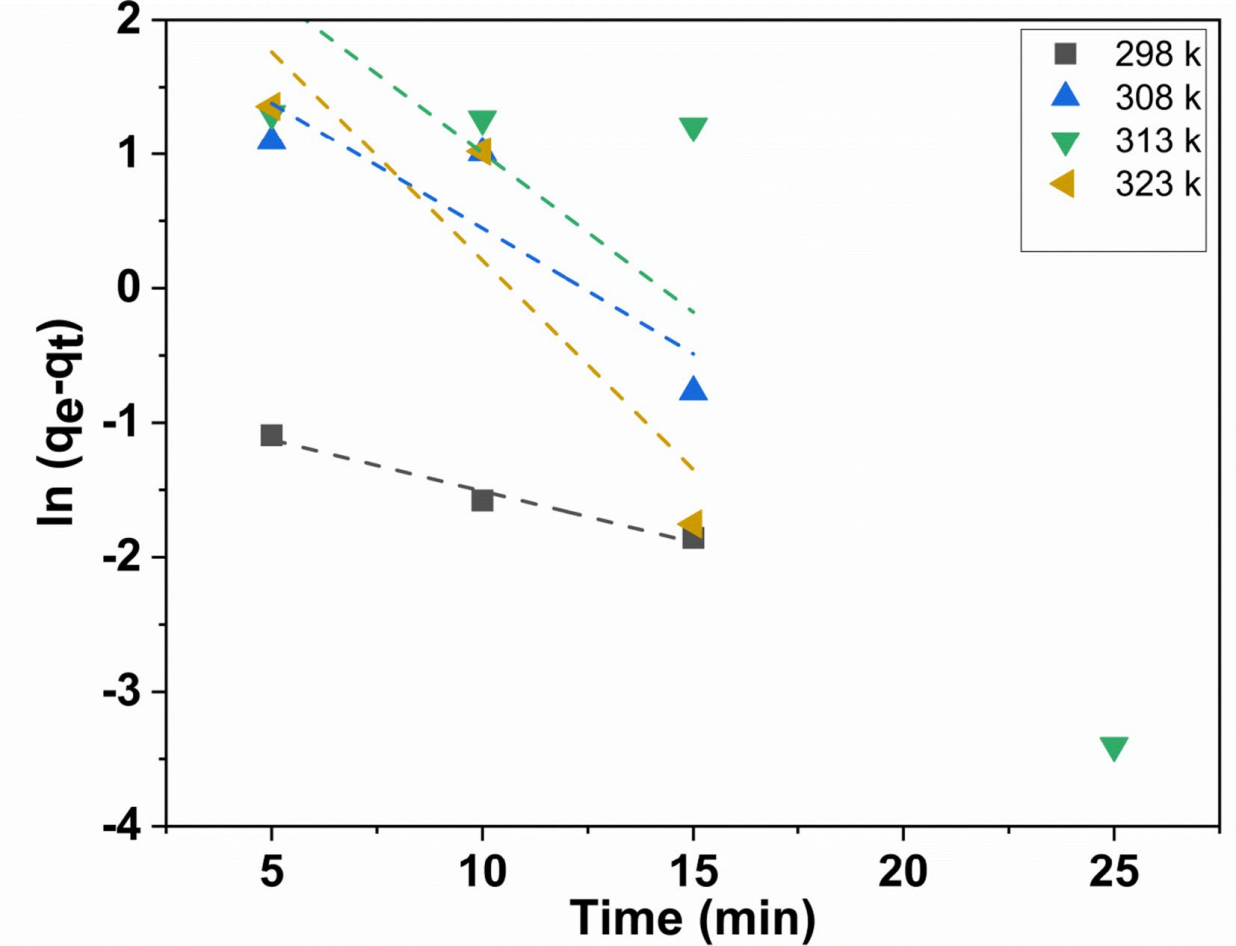
Pseudo-first-order kinetic plots for the adsorption of MB dye at various temperatures.

It is essential to take into account both energy and entropy changes as a fundamental thermodynamic function, in order to verify that any adsorption process occurs spontaneously. To evaluate the thermodynamic characteristics of the adsorption system^48^, an analysis was carried out to ascertain the adsorption of dye at an equilibrium through different temperatures (298, 308, 313, and 323 K), as illustrated in Fig. 11.

The Arrhenius Eq. (4)^8,49^ can be used to compute the activation energy (Ea) for dye adsorption. It displays the Arrhenius-type relationship between temperature and the constant rate of dye adsorption:

10 $$\:{ln\:k}_{2}=\text{ln}A-\:\frac{{E}_{a}}{RT}$$

Where E_a_ represents the Arrhenius activation energy of adsorption; A stands for the Arrhenius factor; R denotes the gas constant, equivalent to 8.314 J mol^−1^ K^−1^; and T represents the operating temperature in Kelvin.

**Fig. 12:**
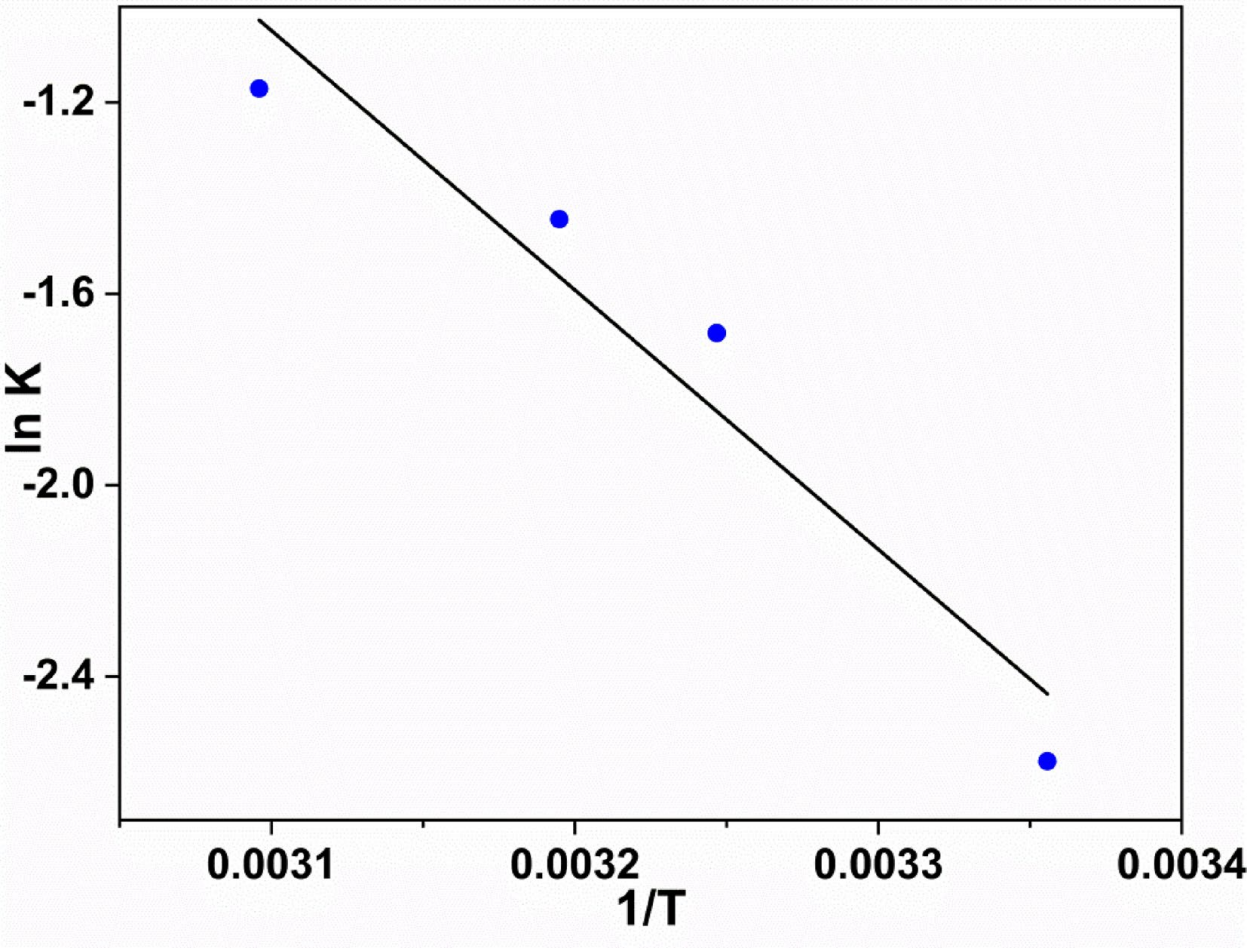
Arrhenius plot for the adsorption of MB dye onto ZrMOF@Bentonite.

When ln k is plotted versus 1/T, Fig. 12 illustrates a good linear relationship between the two variables, as indicated by the correlation coefficient of 0.9267.

Furthermore, the computed activation energy for the adsorption of MB onto ZrMOF@Bentonite was found to be 5.4218 K J.

Going off after, it is essential to access the thermodynamic aspects. Thus several thermodynamic parameters are associated with the temperature dependence of the adsorption process. To determine whether an adsorption process is spontaneous or not. The changes in the free energy (∆G), enthalpy (∆H), and entropy (∆S), which are the thermodynamic parameters.

Eyring’s Eq. (11) can be utilized to calculate the entropy of activation and free energy of activation.

11 $$\:\text{K}=\frac{kT\:\:}{h}{\text{e}}^{\varDelta\:\text{S}/\text{R}}\:{\text{e}}^{-\varDelta\:\text{H}/\text{R}\text{T}}\:$$

In the given equation, K represents the rate constant, k corresponds to the Boltzmann constant (1.380*10^−16^ m^2^ kg sec^−1^ K^−1^), h denotes Plank’s constant (6.626*10^−27^ m^2^ kg sec^−1^), ∆S represents the entropy of activation, ∆H represents the entropy of activation, and T signifies the absolute temperature.

12 $$\Delta H=\Delta E - 2RT$$

13 $$\Delta G=\Delta H - T{\text{ }}\Delta S$$

Inspection of Table 3, one can conclude that the adsorption of MB molecules proceeds spontaneously through a physisorption nature. This can be verified upon assignment of the negative sign of ∆G where ∆S possesses a positive sign, and thus showing the high probability of such adsorption nature.

Table 3 presents the values of ∆G, ∆H, and ∆S for the adsorption of MB onto the ZrMOF@Bentonite surface at different temperatures. Further, the negative values of ∆G indicate the adsorption of MB is stable, highly visible, and spontaneous through the entire temperature range^29,50^, while the positive ∆S value refers to the strong affinity of the MB dye toward the adsorbent surface^29^. On the other hand, the negative ∆H values indicate that the adsorption process is exothermic in nature^51,52^.

**Table 3:** Thermodynamic parameters were calculated for the adsorption process.

Temperature (kelvin)	K (sec^− 1^)	∆E (KJ)	∆H (KJ/mol)	∆S (KJ/mol)	∆G (KJ/mol)
298	0.076	5.421805	-2.482994	3.664 × 10^−3^	-3.574989
308	0.186	5,421,805	-2.566134	3.560 × 10^−3^	-3.662837
313	0.236	5.421805	-2.607704	3.534 × 10^−3^	-3.713852
323	0.31	5.421805	-2.690844	3.504 × 10^−3^	-3.822956

With regard to the effect of temperature, it is well known that evaluating the temperature is beneficial to the discussion of the adsorbate molecules from liquid to solid phases due to the decrease in viscosity of the dye solution. However, from the present study it is noticed that the adsorption capacity and removal rate of MB are gradually decreased with increasing temperature. This can be attributed to the way that high temperatures result in the enhancement of the mobility of the adsorbed MB molecules, and thus giving rise to a noticeable adsorption of MB molecules.

#### Adsorption mechanism

**Fig. 13:**
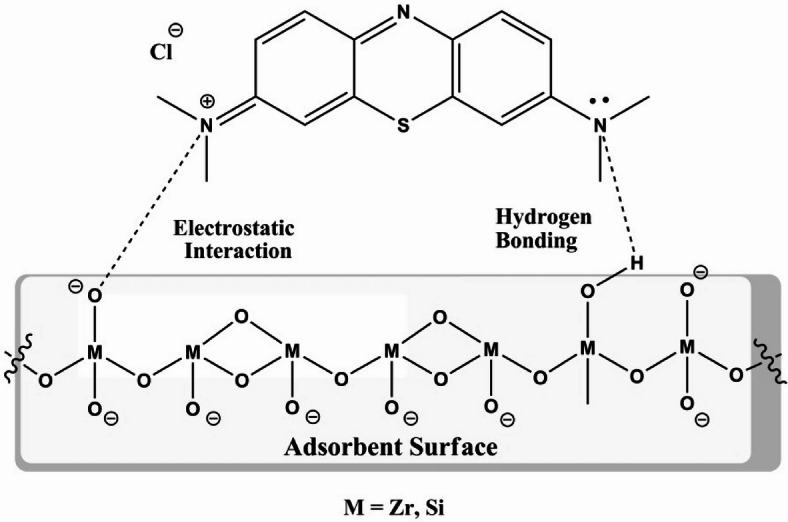
The proposed adsorption mechanism of ZrMOF@Bentonite.

The mechanistic aspects of the removal of methylene blue dye from aqueous solution can be discussed from the above obtained kinetic isotherm data. In this way, the different adsorption mechanisms, including electrostatic forces, hydrogen bonding, and acid-base interactions, have been proposed to interpret the nature of interaction between the synthesized adsorbent surface and MB molecules. Meanwhile, MB dye as cationic organic molecules were adsorbed at the surface of ZrMOF@Bentonite originally through electrostatic forces. This can be attributed to the presence of various carboxylated groups coordinated with both zirconium and the different metal silicates in bentonite catalysts. In addition, the trend of variation of pH together with the already deprotonated metal-oxygen species on the surface, could lead us to suggest that there are essentially two coexisting types of interaction, either electrostar attraction and/or via hydrogen bonding. Accordingly, the possible proposed adsorption mechanism can be illustrated in Fig. 13.

### Adsorbent stability and reusability

**Fig. 14:**
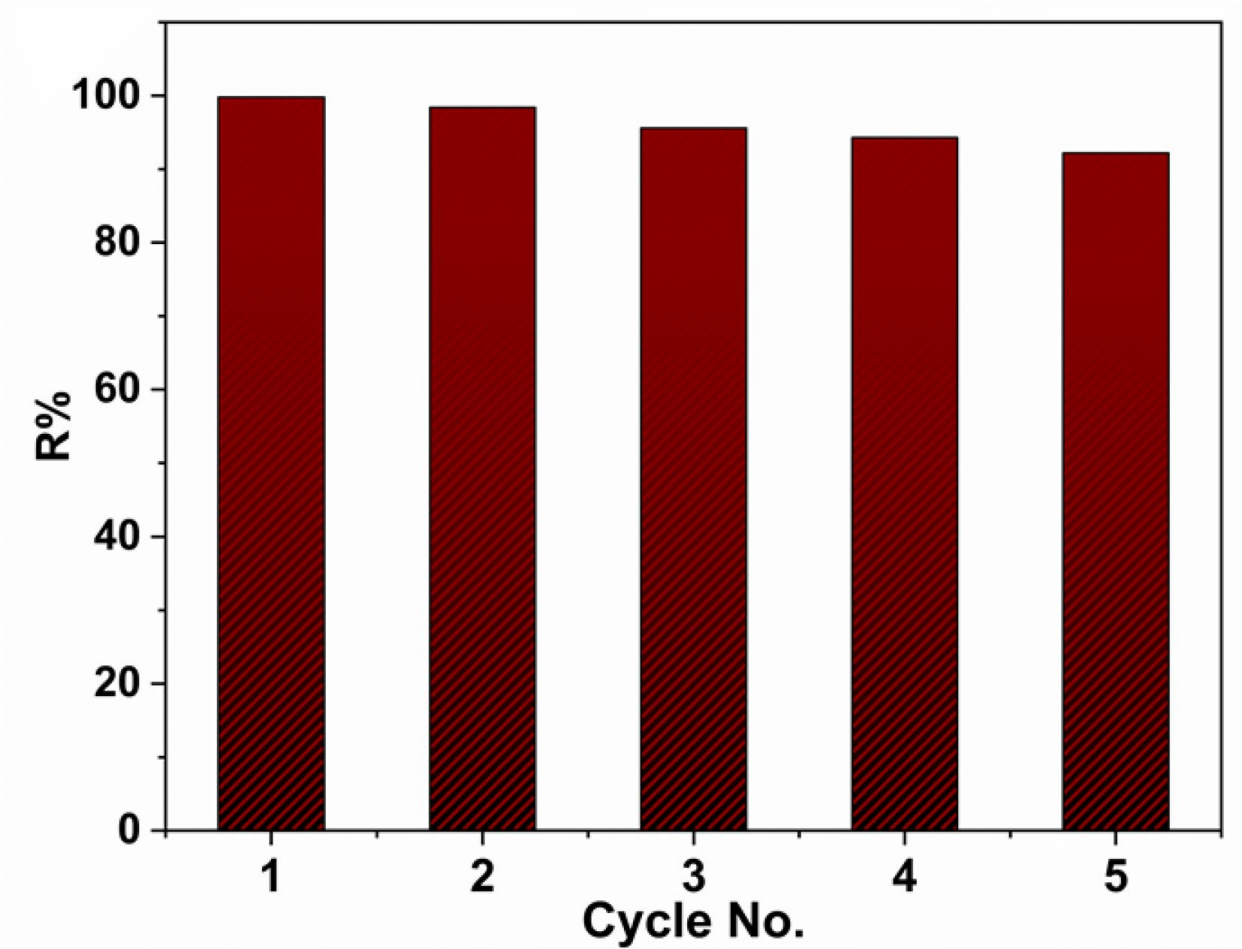
Reusability of ZrMOF@Bentonite in the adsorption of MB dye at room temperature.

The reusability of the adsorbent is very vital for its practical application in adsorption dye from wastewater. To examine the reusability of the adsorbent in the adsorption of methylene blue dye (100 mg/L) with the adsorbent dose (50 mg), the adsorbent was separated after the adsorption process at room temperature, washed with distilled water, and then dried overnight to recover it for further cycles. The reusability test of our adsorbent is given in Fig. 14. It is clear from the figure that the removal percent of dye decreases slightly as the adsorbent is reused. This decrease can be attributed to the active sites on the adsorbent surface becoming filled with dye molecules. Therefore, the adsorbent activity continuously declines with use in the further 5 runs.

## Conclusion

In this work, the natural bentonite sample collected from Abu Tartur area, Western Desert, Egypt was modified through a sustainable synthesis of new nanocrystalline material by using metals organic framework MOF (Zr BDC). The obtained composite was characterized by various physicochemical techniques. The porous material is applied as an eco-friendly and novel adsorbent for the removal of MB dye from aqueous solutions. The adsorbent has been demonstrated to be highly effective for the adsorption of the cationic dye MB from the solution. It is noted that this adsorption is positively affected when adsorbent dose, pH, dye concentration, and time of contact were augmented 0.025–0.17 g, 2–6, 4.7–28.9 mg/g, 10–100 mg/l, and 0–180 min respectively. Additionally, it is found that the adsorption was positively influenced when pH increases in the range of 2–6. The kinetic data and isothermal results proved that this described adsorption will be clarified by using kinetic models viz. intra-particle diffusion, pseudo first and second orders, and Elovich ones. Furthermore, inspection of the isothermal data showed that the Langmuir and Freundlich models have a higher correlation coefficient (R^2^ = 0.97). On the other hand, it is found that the maximum monolayer capacity for MB adsorption amounted to 32.2 mg/g. From the above quantitative results, it can be mentioned that the composite is quite an effective adsorbent for the removal of MB from an aqueous solution and has a good potentiality for further applications in the future.

From the kinetic study it is found that it appropriately obeys pseudo-second-order kinetics with a high correlation coefficient (R2 = 0.97) and exhibit a multi-step diffusion process. The acidic conditions result in an enhancement of dye removal, which can be attributed to the presence of both electrostatic attraction and hydrogen bonding, moreover, the thermodynamic calculation indicates a negative sign of ∆G where the positive sign corresponds to ∆S, and so referring that the adsorption of MB onto the adsorbent surface was stable, highly feasible, and spontaneous. Finally, the observed exothermic behavior from the negative sign of ∆H (2.69 KJ/mol). Therefore, this study prevails that the already synthesized composite is a readily available and environmentally friendly adsorbent and might be applied as a novel promising and non-conventional adsorbent for removing dye from polluted water.

## Data Availability

the data and chemical analysis that represent in the research article are available with the corresponding author (Fatma M. Dardir) in reasonable request.
